# Supplementary material for: Virulence Characterization of Puccinia striiformis f. sp. tritici in China in 2020 Using Wheat Yr Single-Gene Lines
Source: J Fungi (Basel). 2025 Jun 12;11(6):447. doi: 10.3390/jof11060447 (PMC12194282; doi:10.3390/jof11060447)
Supplement: Supplementary file 1 [file jof-11-00447-s001.zip › jof-3669870-supplementary.pdf]

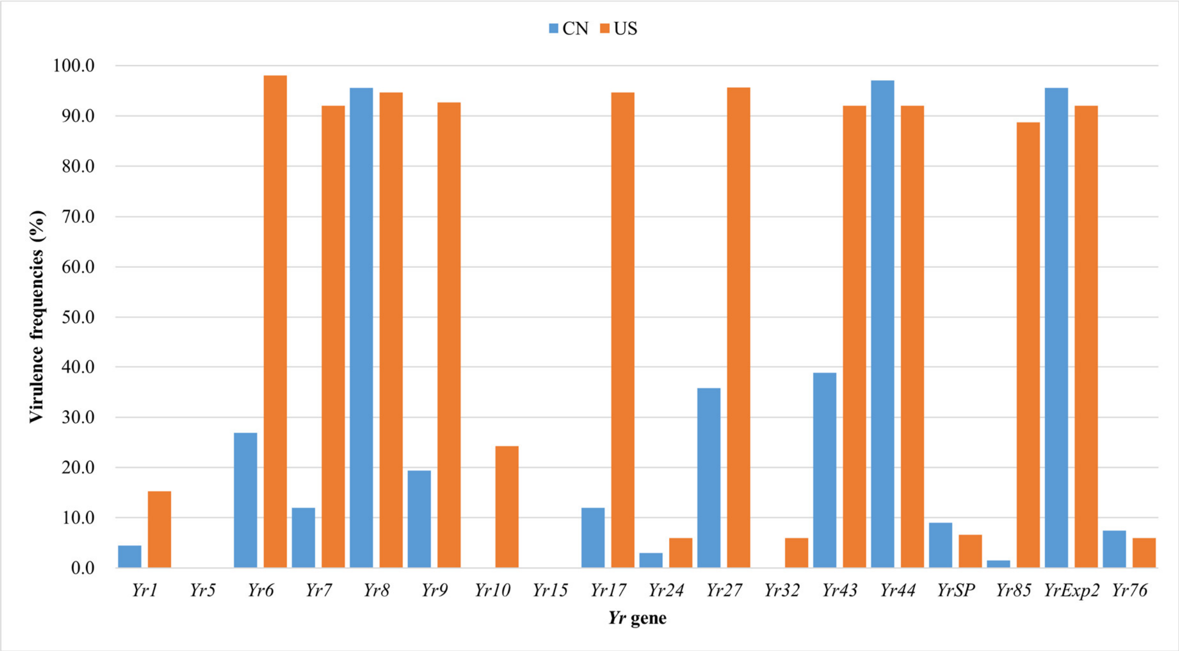

Figure S1. The virulence frequencies of *Puccinia striiformis* f. sp. *tritici* (*Pst*) between China and the United States in 2020. The virulence of *Pst* races in the U.S. were from https://striperust.wsu.edu (accessed on 20 September 2022).
